# 5-aminolevulinic acid (ALA) deficiency causes impaired glucose tolerance and insulin resistance coincident with an attenuation of mitochondrial function in aged mice

**DOI:** 10.1371/journal.pone.0189593

**Published:** 2018-01-24

**Authors:** Shinichi Saitoh, Satoshi Okano, Hidekazu Nohara, Hiroshi Nakano, Nobuyuki Shirasawa, Akira Naito, Masayuki Yamamoto, Vincent P. Kelly, Kiwamu Takahashi, Tohru Tanaka, Motowo Nakajima, Osamu Nakajima

**Affiliations:** 1 Research Center for Molecular Genetics, Institute for Promotion of Medical Science Research, Yamagata University Faculty of Medicine, Yamagata, Yamagata, Japan; 2 Department of Anatomy and Structural Science, Yamagata University Faculty of Medicine, Yamagata, Yamagata, Japan; 3 Department of Medical Biochemistry, Tohoku University School of Medicine, Sendai, Japan; 4 School of Biochemistry & Immunology, Trinity Biomedical Sciences Institute, Trinity College Dublin, Dublin, Ireland; 5 SBI Pharmaceuticals Co., Ltd., Tokyo, Japan; Hungarian Academy of Sciences, HUNGARY

## Abstract

In vertebrates, the initial step in heme biosynthesis is the production of 5-aminolevulinic acid (ALA) by ALA synthase (ALAS). ALA formation is believed to be the rate-limiting step for cellular heme production. Recently, several cohort studies have demonstrated the potential of ALA as a treatment for individuals with prediabetes and type-2 diabetes mellitus. These studies imply that a mechanism exists by which ALA or heme can control glucose metabolism. The *ALAS1* gene encodes a ubiquitously expressed isozyme. Mice heterozygous null for *ALAS1* (*A1*^*+/-*^s) experience impaired glucose tolerance (IGT) and insulin resistance (IR) beyond 20-weeks of age (aged *A1*^*+/-*^s). IGT and IR were remedied in aged *A1*^*+/-*^s by the oral administration of ALA for 1 week. However, the positive effect of ALA proved to be reversible and was lost upon termination of ALA administration. In the skeletal muscle of aged *A1*^*+/-*^s an attenuation of mitochondrial function is observed, coinciding with IGT and IR. Oral administration of ALA for 1-week brought about only a partial improvement in mitochondrial activity however, a 6-week period of ALA treatment was sufficient to remedy mitochondrial function. Studies on differentiated C2C12 myocytes indicate that the impairment of glucose metabolism is a cell autonomous effect and that ALA deficiency ultimately leads to heme depletion. This sequela is evidenced by a reduction of glucose uptake in C2C12 cells following the knockdown of *ALAS1* or the inhibition of heme biosynthesis by succinylacetone. Our data provide *in vivo* proof that ALA deficiency attenuates mitochondrial function, and causes IGT and IR in an age-dependent manner. The data reveals an unexpected metabolic link between heme and glucose that is relevant to the pathogenesis of IGT/IR.

## Introduction

5-aminolevulinic acid (ALA) production is the first step in heme biosynthesis, which in higher vertebrates is a function of the ALA synthase (ALAS) enzyme. In most cell types, ALA production is considered the rate-limiting step in cellular heme biosynthesis [1]. Therefore, a shortage of cellular ALA can lead to insufficient heme levels. Two ALAS isozymes exist, which are encoded on separate chromosomes [2]. *ALAS1* is expressed in a ubiquitous manner [3] and supplies “housekeeping heme” for numerous hemoproteins; including those of the mitochondrial electron transport chain (ETC). *ALAS2* is expressed preferentially in erythroid cells [4,5] and is essential for the biosynthesis of bulk heme for hemoglobin production [6–8].

Previously, we established *ALAS1*-null mice and observed embryonic lethality by E7.5, suggesting that *ALAS1* is essential for early development [9], and supporting the theory that ubiquitous *ALAS1* is indispensable for heme supply in most tissues. By contrast, *ALAS1* heterozygous mice (designated as *A1*^+/-^s below) were obtained according to Mendelian ratios and developed to adulthood, despite expressing only 50% the control level of *ALAS1* mRNA in liver [9].

In all vertebrate tissues, heme acts as a cofactor for enzymes of the mitochondrial ETC including Complex II-IV and cytochrome c [10]. In fact, it has been observed that heme deficiency can interrupt the assembly of mitochondrial Complex IV in human fibroblasts [11]. It remains unclear however, whether heme deficiency *in vivo* leads to mitochondrial dysfunction.

Two recent cohort studies in Japan [12] and Hawaii [13], suggest that oral ALA can protect against mild hyperglycemia and help prevent type-2 diabetes mellitus (T2DM). These studies strongly suggest that heme or ALA is associated with glucose metabolism *in vivo*. Conversely, glucose is known to influence heme metabolism. Acute attacks of inducible hepatic porphyria can be treated by high glucose load. This beneficial effect of glucose is thought to act through the down-regulation of *ALAS1* by the peroxisome proliferator-activated receptor ɤ coactivator 1α (PGC1α) [14].

The transcriptional repressor Rev-erbα possesses a heme binding domain that is essential for its repressor activity [15–17]. In liver cells, heme-sensing by Rev-erbα acts to regulate glucose homeostasis (by suppressing glucose output and the expression of gluconeogenic genes such as glucose 6-phosphatase (*G6Pase*) and phosphoenolpyruvate carboxykinase (*PEPCK*)), to control circadian rhythms (by regulation of the *Bmal1* gene) and to modulate energy metabolism (by, *inter alia*, the supply of heme for mitochondrial respiration). Using human HepG2 liver cells, it has been shown that the expression of Rev-erbα-target genes—such as the gluconeogenic genes *G6Pase* and *PEPCK*—are repressed by heme repletion and activated by *ALAS1* knockdown [17]. The physiological significance of gluconeogenic control by Rev-erbα remains unclear however, since previous studies on *Rev-erbα*^-/-^ mice reported no remarkable abnormalities in glucose metabolism [18–20]. It is possible that heme deficiency *in vivo* impairs gluconeogenesis through Rev-erbα transcriptional dysregulation [19,20].

To clarify the relationship between heme and glucose metabolism *in vivo* and to understand further the molecular mechanism, we examined glucose metabolism in *A1*^+/-^s. Here we show that *A1*^+/-^s experience impaired glucose tolerance (IGT) and insulin resistance (IR). These symptoms can be reversed by ALA administration for 1 week (wk). In the skeletal muscle of *A1*^+/-^s an attenuation of mitochondrial function was observed. In contrast to the effect on IGT and IR, only a partial recovery in mitochondrial function was achieved by a 1 wk treatment of ALA and instead, a prolonged treatment period of 6 wks was required to elicit a positive effect.

## Materials and methods

### Mice

*ALAS1* heterozygous mice (*A1*^+/-^s) were generated on a mixed background, 129Sv/C57BL/6, as previously described [9]. *A1*^+/-^s animals were maintained by crossbreeding to BDF1 (F1 hybrid of C5BL/6 and DBA2) with male *A1*^+/-^s and wild-type littermates (WTs) used for experiments. Mice 8–12 wk-old or 18–35 wk-old were classed as young *A1*^+/-^s/WTs and aged *A1*^+/-^s/WTs, respectively. Mice were housed in a 14h -10h light-dark cycle and allowed access to regular chow diet and water *ad libitum*. All animal studies were conducted in accordance with The Regulation of Animal Experiments in Yamagata University and approved by The Institutional Animal Care and Use Committee of Yamagata University (Approved Number 29–004).

### Genotyping

The genotypes of mice were determined by PCR using the three primers: 5’-ACAACCACTACCTGAGCACCCAG-3’, 5’-AGAGTGTGGCTCCCATGT-3’ and 5’-GCTGGAGGGGTTTCTTTGACC-3’, that permitted the detection of the *ALAS1*-targeted allele (835bp) and wild-type allele (348bp).

### Quantification of daily amounts of excreted ALA in urine

Urine was collected for 5 days in metabolic cages. The concentration of urinary ALA was determined by a modification of Morita’s method [21] and Oishi’s method [22]. Briefly, 50 μl of urine was added to 1.75 ml of acetylacetone reagent (acetylacetone-ethanol-water 3:2:15, v/v/v) and 225 μl 10% formaldehyde aqueous solution and heated to 100°C for 10 min. After cooling, the reaction mixture was filtered through a disposable HPLC filter (13 mm diameter, 0.45 μm pore size; Merck Millipore) and products resolved by high-performance liquid chromatography (LC-2000 series; JASCO) on an ODS reserved-phase silica column (TSKgel ODS-80Tm, TOSOH; 150 x 4.6 mm). The flow rate, oven temperature, and detector wavelength were set at 0.75 ml/min, 40°C, and 373/463 nm (excitation/emission), respectively. The amount of daily urinary ALA excreted was calculated from the daily means of ALA in the urine collected over a 1 wk period.

### ALA administration

For ALA supplementation, oral administration was performed on aged *A1*^+/-^s (300 mg/kg BW) once each day for 1 wk. To evaluate the effect of ALA administration over time, 200 mg/kg BW was administrated for 1 or 2 wks. Also, to evaluate the dose-dependency of ALA, 200 or 400 mg/kg BW was administrated for 1 wk. Oral administration of ALA for 3 and 6 wks was conducted by addition to the drinking (1.5 mg/ml) to avoid long-term stress from the feeding needle. Mice typically drank 6 ml of water per day to give a daily intake of 9 mg ALA (300 mg/kg BW in the case of a 30 g mouse).

### Measurement of blood glucose, serum insulin, serum glucagon, serum iron, serum ferritin levels and total iron binding capacity (TIBC)

Blood glucose levels were measured with an ACCU-CHEK aviva meter (Roche Diagnostics). Serum insulin levels were measured using the Mouse Insulin ELIZA Kit (Morinaga Institute of Biological Science). Serum glucagon levels were measured using a Bio-Plex system (Bio-Rad laboratories). Serum ferritin levels were measured using the mouse ferritin ELIZA kit (Abcam). Serum iron levels and TIBC were measured according to the Nitroso-PSAP Method [23]. Unsaturated iron-binding capacity (UIBC) was calculated by the difference between serum iron and TIBC.

### Fluorometric assay of heme in the mitochondrial fraction of skeletal muscle

Heme content was quantified as previously described [24]. The mitochondrial or cytosolic fraction of skeletal muscle was added to 50 vol. of 1 M oxalic acid and immediately heated for 2 hrs at 100°C. After cooling, fluorescence was determined in a microplate reader (Ex 400 nm/ Em 622 nm).

### Oral glucose tolerance tests (OGTT) and insulin tolerance tests (ITT)

In OGTT, each mouse was fasted overnight and administered glucose orally (1 mg/g BW) [25]. In ITT, each mouse was fasted for a short period (about 1 h) and then intraperitoneally injected with human insulin (1 mU/g BW, Novo nordisk). Blood was taken from the from tail vein for measurements of blood glucose or serum insulin levels before and 15, 30, 60, 120 mins after administration of glucose or insulin.

### Gene expression analysis

Total RNA was isolated from tissue and cells using ISOGENE (Nippon Gene). Isolated RNA was reverse-transcribed into cDNA using SuperScript II Reverse Transcriptase (Life Technologies). Quantitative RT-PCR analyses were performed using the SsoAdvanced Universal SYBR Green Supermix with a CFX96 Real-Time PCR Detection System (Bio-Rad). Expression levels were calculated using the comparative critical threshold (Ct) method. Each reaction was run in triplicate or duplicate using specific primer sets (Table 1).

**Table 1:** List of specific primers used for real-time PCR analyses.

Gene	Forward primer (5’->3’)	Reverse primer (5’->3’)
***Alas1***	GTCGGTTTAGCGTCCTCCGCTCGAGT	GCAGTGGACAGCTGATGTGACAGGG
***Glut4***	CCTCTACATCATCCGGAACC	ACATTGGACGCTCTCTCTCC
***PGC-1α***	AATCAGACCTGACACAACGC	GCATTCCTCAATTTCACCAA
***G6Pase***	CGACTCGCTATCTCCAAGTGA	CGACTCGCTATCTCCAAGTGA
***PEPCK***	GCAACTTAAGGGCTATCAACC	CGGTCTCCACTCCTTGTTC
***G3PDH***	GGCAAAGTGGAGATTGTTGC	TGGTGAAGACACCAGTAGACTCC
***β-actin***	TCACCCACACTGTGCCCATCTACGA	CAGCGGAACCGCTCATTGCCAATGG
***mt-Nd1*(mt)**	CAGCCTGACCCATAGCCATA	ATTCTCCTTCTGTCAGGTCGAA
***n-β Globin (nuclear)***	GAAGCGATTCTAGGGAGCAG	GGAGCAGCGATTCTGAGTAG

### Western blot

Whole protein extracts were prepared from quadricep muscles and cells by homogenization in RIPA buffer containing protease inhibitors (cOmplete™ Protease Inhibitor Cocktail, Roche) and phosphatase inhibitors (PhosSTOP™ Phosphatase inhibitor cocktail, Roche). Homogenates were centrifuged and the supernatant was collected. Protein extracts were quantified and an equal amount of protein was loaded onto an SDS-polyacrylamide gel. The gel was blotted on a polyvinylidene Fluoride (PVDF) microporous membrane (Millipore). Membranes were blocked in Tris Buffered Saline with 0.1% Tween 20 (TBST) containing 2% BSA. Primary antibody reactions were performed using antibodies against the following proteins: ALAS1 (kindly gifted from Prof. Munakata in Kinki University School of Medicine, Japan), Akt, p-Akt (Thr308), p-Akt (Ser473), IRS-1, HexokinaseⅡ, and α-Tubulin (Cell Signaling Technology), IRS1 (phosphor Y612) and Glut4 (Abcam), PGC-1α and GAPDH (Santa Cruz), α-Actinin (Sigma-Aldrich), UQCRC2 (Abcam), REVERBα (PPMX) and MTCO1 (COX IV) (Abcam). Secondary antibodies used were either HRP-linked anti-mouse or anti-rabbit antibodies (Cell signaling Technology). Immunocomplexes were detected by ECL Prime Western Blotting Detection Reagent (GE Healthcare) and optical densities were measured using Light-Capture and CS Analyzer software (ATTO).

### Transmission electron microscopic (TEM) examination

Samples were fixed with 2% paraformaldehyde (PFA) and 2% glutaraldehyde (GA) in 0.1 M phosphate buffer (PB) pH 7.4 at 4°C overnight. After fixation, the samples were washed 3 times with 0.1 M PB for 30 min. each, postfixed with 2% osmium tetroxide (OsO_4_) in 0.1M PB at 4°C for 2 h, and dehydrated in graded ethanol solution (50–100%). The samples were infiltrated with propylene oxide (PO) two times for 30 min. each and were put into a 70:30 mixture of PO and resin (Quetol-812; Nisshin EM Co., Tokyo, Japan) for 1 h, PO was volatilized overnight. The samples were transferred to fresh 100% resin, and polymerized at 60°C for 48 h. The polymerized resins was sectioned to a thickness of 70 nm, and sections mounted on copper grids, stained with 2% uranyl acetate at room temperature (RT) for 15 min., then washed with distilled water followed by a secondary-stain with Lead stain solution (Sigma-Aldrich) at RT for 3 min. The grids were observed by transmission electron microscope (JEM-1400Plus; JEOL Ltd., Japan) at an acceleration voltage of 80 kV. To estimate the size of mitochondria, the short and long diameters were measured on either side of the electron-opaque Z line in skeletal muscle in TEM images.

### Histology and immunohistochemistry

Skeletal muscle was frozen in isopentane cooled with liquid nitrogen. Cross-sections of skeletal muscle were incubated with antibody against GLUT4 (1:1000, Abcam). After incubation with the fluorescent-labeled secondary antibody (goat anti-rabbit IgG conjugated to AlexaFluor 546, Invitrogen) and DAPI (Wako), sections were mounted in DAKO. Paraffin-embedded sections were stained with Prussian blue to examine the presence of ferric iron as previously described [6].

### Cell culture

C2C12 cells and Hepa1-6 cells were maintained in high-glucose (4500 mg/L) Dulbecco’s Modified Eagle Medium (D-MEM; Sigma-Aldrich) supplemented with 10% fetal bovine serum. Cells were grown at 37°C in 5% CO_2_. *ALAS1* knock-down and control C2C12 cells were established by stable transfection of an expression plasmid encoding *ALAS1* shRNA or scrambled control shRNA. C2C12 myocyte differentiation was induced by incubation of the cells in DMEM supplemented with 2% horse serum for 4 days [26]. In case of ALA treatment, C2C12 cells were incubated in DMEM supplemented with 2% horse serum for 3 days, followed by incubation with 100 μM ALA in DMEM supplemented with 2% horse serum for 1 day.

### Measurement of insulin-induced glucose uptake

Insulin-induced glucose uptake was measured in differentiated C2C12 cells using a 2-Deoxyglucose (2-DG) Uptake Measurement Kit (Cosmo Bio Co., LTD, Tokyo, Japan). Differentiated C2C12 myocytes were incubated in PBS for 15 min to reduce cellular glucose. Subsequently, cells were incubated with or without 10 μM cytochalasin B (Wako Pure Chemical Industries, Japan) in serum-free low-glucose (1000mg/L) DMEM (Sigma-Aldrich) for 15 min, and then, treated with 500 nM bovine insulin (Sigma-Aldrich) for 15 min, followed by incubation with 1 mM 2-DG for 30 min. The cells were lysed to measure 2-deoxyglucose 6-phosphate. The amount of insulin-induced glucose uptake was calculated as the difference in total glucose uptake with or without cytochalasin B; an inhibitor of insulin-stimulated glucose uptake [27]. The amount of cellular glucose uptake was normalized to protein concentration. The amount of insulin-independent glucose uptake of both control- and ALAS1- shRNA C2C12 myocytes was approximately 0.01 μmol/g protein in every experiment.

### Measurement of mitochondrial DNA levels

Total DNA isolated from tissue was used as template. Mitochondria DNA (mtDNA) copy number was estimated by the ratio of nuclear to mitochondrial gene levels by real-time PCR analyses using the following primer pairs: *Nd1* for mtDNA and *β-Globin* for nuclear DNA (Table 1). Comparative Ct values were used to quantify the relative amounts of mtDNA to nuclear DNA [28, 29].

### Treadmill test

Physical endurance was evaluated using a treadmill for Rats and Mice (MK-690S; Muromachi Kikai). Before the exercise stress test, mice were practiced for 2 days to adapt to the equipment and the electrical stimulation. On the day of the exercise stress test, mice were forced to run on the treadmill at a speed of 11 m/min at an incline of 15%. The treadmill speed was increased by 1 m/min every 2 min, up to 20 m/min. The running distances were determined until exhaustion when mice continuously touched the electrical grid. Before and after running, blood lactate levels were measured using Lactate Pro 2 (Arkray).

## Results

### Reduced *ALAS1* expression in insulin-target organs of *A1*^+/-^ mice

**Fig 1:**
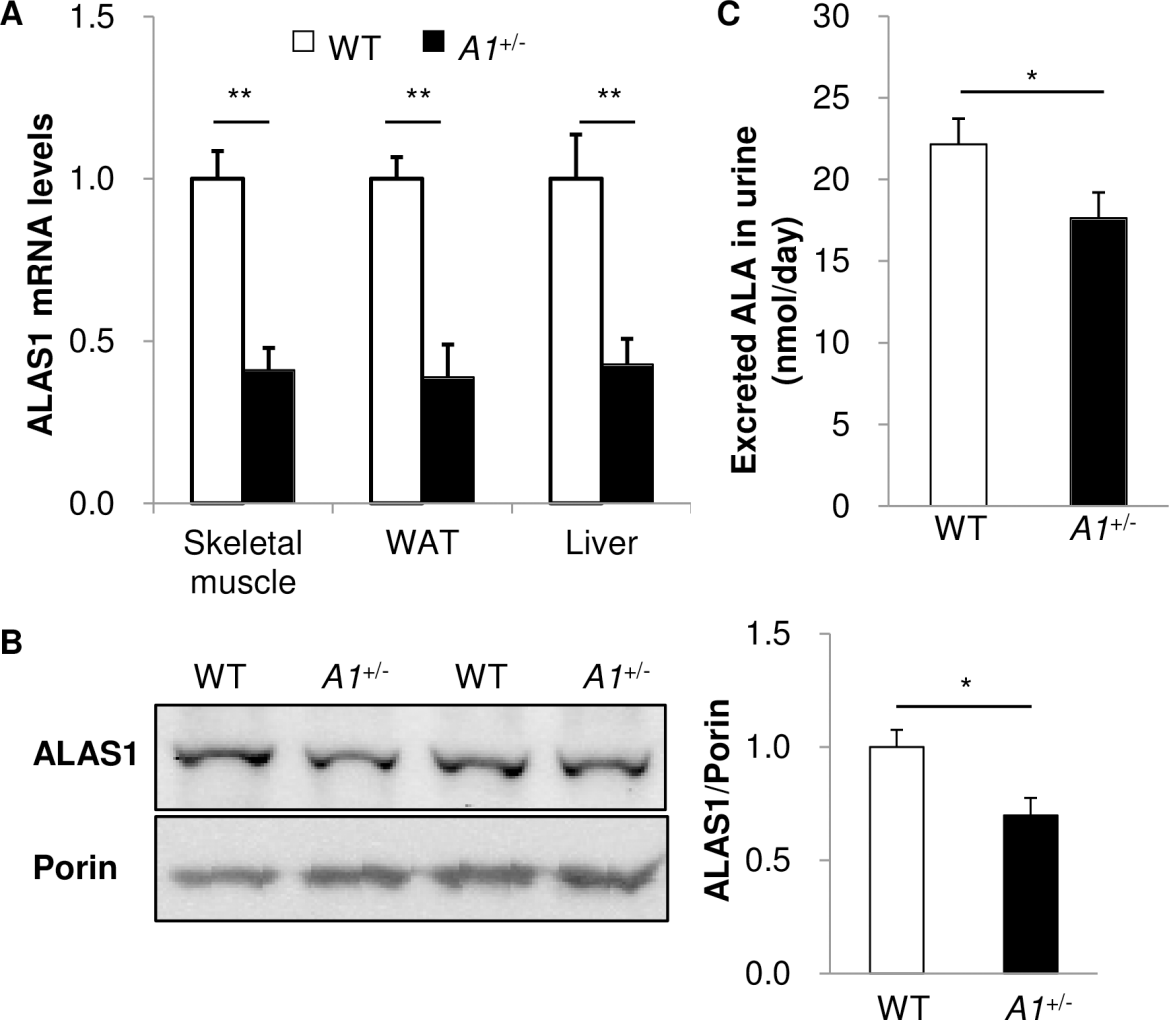
Deficiency of *ALAS1* mRNA and ALA in aged *A1*^+/-^s. (**A**) *ALAS1* mRNA expression levels in insulin target tissues (skeletal muscle, white adipose tissue (WAT) and liver) of aged WTs and *A1*^+/-^s (n = 3–4 each genotype). (**B**) ALAS1 protein expression in mitochondrial fractions of skeletal muscle from aged WTs and *A1*^+/-^s (n = 5 per group). (**C**) Daily amounts of excreted ALA in the urine of aged WTs and *A1*^+/-^s (n = 4 per group). Values are means ± s. e. m. for the indicated number of measurements. Statistical significance was determined by 2-tailed unpaired Student’s *t*-test, *P<0.05, **P<0.01.

The levels of *ALAS1* mRNA in insulin-target tissues—skeletal muscle, white adipose tissue, and liver—of 18–35 wk-old *A1*^+/-^s (designated as aged *A1*^+/-^s) were less than half that of wild-type controls (aged WTs) consistent with gene dosage (Fig 1A). Mitochondrial fractions from the skeletal muscle of aged *A1*^+/-^s showed reduced ALAS1 protein levels compared to aged WTs (Fig 1B). Concordantly, reduced daily amounts of excreted ALA were detected in the urine of aged *A1*^+/-^s (Fig 1C), suggesting that aged *A1*^+/-^s show a systemic reduction in ALA production. However, we detected no significant change between heme content in mitochondrial or cytosolic fractions from the skeletal muscle of aged *A1*^+/-^s and aged WTs (S1 Fig in Supporting Information). In addition, serum iron levels, total iron binding capacity (TIBC), unsaturated iron binding capacity (UIBC) and serum ferritin levels in aged *A1*^+/-^s were similar to those of aged WTs (S2A–S2D Fig in Supporting Information). Finally, we found no notable iron deposition in skeletal muscle and liver of both aged mice in histological examination with Prussian blue (data not shown).

### Aged *A1*^+/-^s show ALA-dependent impaired glucose metabolism

**Fig 2:**
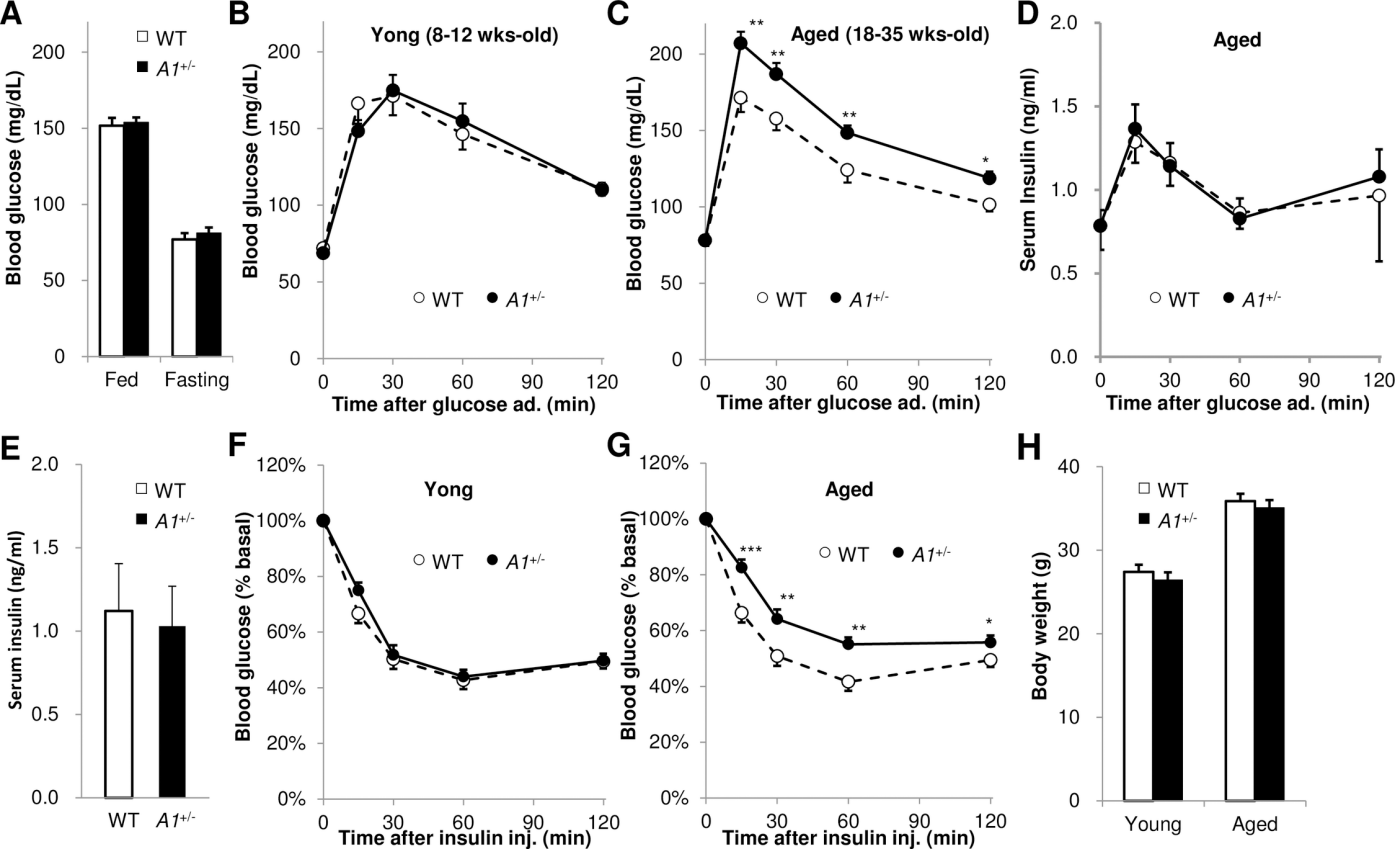
*A1*^+/-^s manifest impaired glucose tolerance and insulin resistance upon maturation. (**A**) Blood glucose levels of fed and fasted aged WTs (n = 24) and *A1*^+/-^s (n = 37). (**B, C**) Oral glucose tolerance test results (OGTT) for young (**B**) and aged (**C**) WTs and *A1*^+/-^s (**B**; n = 10, **C**; n = 20–24 per group). (**D**) Serum insulin levels of aged WTs and *A1*^+/-^s during OGTT (n = 8 per group). (**E**) Serum insulin levels of aged WTs and *A1*^+/-^s under normal fed conditions (n = 10 per group). (**F, G**) Insulin tolerance test (ITT) on young (**F**) and aged (**G**) WTs and *A1*^+/-^s (**F, G**; n = 12–15 per group). (**H**) Body weight of young- and aged WTs and *A1*^+/-^s (n = 24–35 per group). Values are means ± s. e. m. for the indicated number of measurements. Statistical significance was determined by 2-tailed unpaired Student’s *t*-test, *P<0.05, **P<0.01, *** P<0.001.

Aged *A1*^+/-^s showed normal fed and fasted blood glucose levels (Fig 2A). Oral glucose tolerance tests (OGTT) carried out on 8–12 wk-old *A1*^+/-^s, (termed young-*A1*^+/-^s) revealed normal blood glucose levels (Fig 2B). By contrast, similarly challenged 18–35 wk-old *A1*^+/-^s (termed aged *A1*^+/-^s) showed impaired glucose tolerance, with increased blood glucose levels compared with age-matched WTs (termed aged WTs) at all time points measured (Fig 2C). Interestingly, serum insulin levels in aged *A1*^+/-^s were similar to aged WTs when subjected to OGTT (Fig 2D) as well as under normal fed conditions (Fig 2E). Insulin tolerance tests (ITT) revealed that the reduction in blood glucose levels after intraperitoneal administration of insulin was normal in young *A1*^+/-^s (Fig 2F), but impaired in aged *A1*^+/-^s, the latter showing elevated blood glucose levels at all time points measured (Fig 2G). These results suggest that *A1*^+/-^s acquire impaired glucose tolerance and insulin resistance in an age-dependent manner. In all cases, the body weights of young and aged *A1*^+/-^s were similar to those of WTs (Fig 2H).

**Fig 3:**
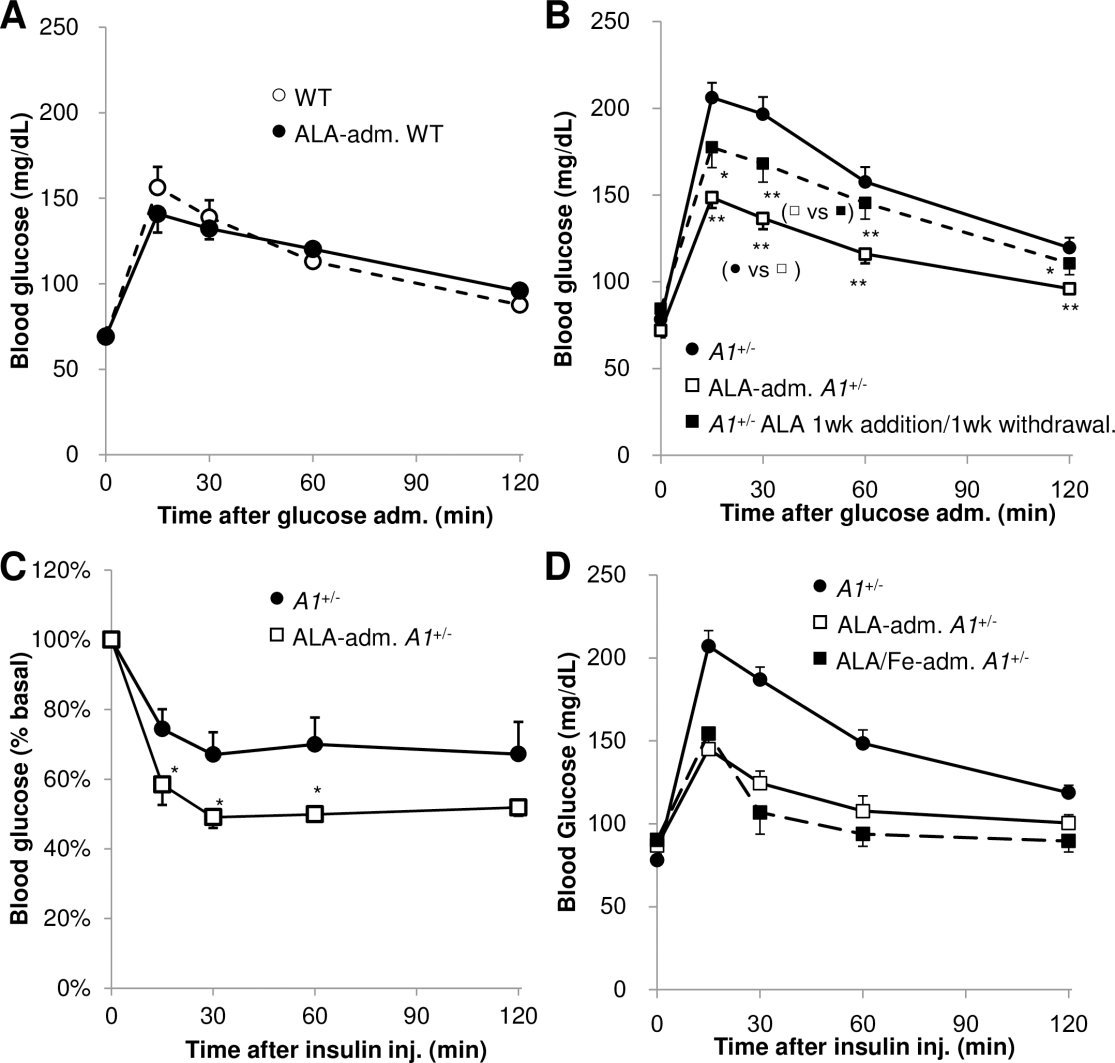
Oral administration of ALA for 1 wk ameliorates impaired glucose tolerance and insulin resistance in aged *A1*^+/-^s. OGTT (**A, B**) and ITT (**C**) after oral administration of ALA (300 mg/kg BW/day) for 1 wk (**A**) to aged WTs (ALA-adm. WT), (**B, C**) to *A1*^+/-^s (ALA-adm. *A1*^+/-^) and (**B**) to *A1*^+/-^s followed by withdrawal of ALA for 1 wk (*A1*^+/-^ ALA 1wk addition/1wk withdrawal) (**A, C**; n = 5, **B**; n = 11 per group). (**D**) OGTT after oral administration of ALA (200 mg/kg BW/day) and ferric citrate (31 mg/kg BW/day) for 1 wk to aged *A1*^+/-^s (ALA/Fe-adm. *A1*^+/-^) (*A1*^+/-^ n = 25, ALA-adm. *A1*^+/-^ n = 5, ALA/Fe-adm. *A1*^+/-^ n = 4). Values are means ± s. e. m. for the indicated number of measurements. Statistical significance was determined by 2-tailed unpaired Student’s *t*-test, *P<0.05, **P<0.01.

Next, the effect of ALA treatment on impaired glucose tolerance and insulin resistance in both aged WT and *A1*^+/-^ animals was investigated. ALA was orally administered to mice for 1 wk before OGTT or ITT. ALA administration (300mg/kg BW/day) for 1 wk had no significant effect on blood glucose levels of WTs after glucose load (Fig 3A), but reduced blood glucose levels in *A1*^+/-^s to normal levels (Fig 3B). Extending the duration of ALA administration to 2-wks (200mg/kg BW/day) or increasing the amount given for 1 wk (400mg/kg BW/day) similarly improved impaired glucose tolerance of *A1*^+/-^s (S3A and S3B Fig in Supporting Information). The insulin resistance phenotype of *A1*^+/-^s was also improved by ALA treatment for 1 wk (300mg/kg BW/day) (Fig 3C) and by treatment with a lower dose of ALA for 1 wk (200mg/kg BW/day) (S3C Fig in Supporting Information).

Appropriate levels of heme biosynthesis rely on the provision of sufficient iron, leading us to speculate that ferric citrate may enhance the effect of ALA when co-administered. However, co-treatment showed no remarkable change over ALA administration alone (Fig 3D). Notably, it was observed that the beneficial effects of ALA on impaired glucose tolerance of *A1*^+/-^s was reversible since the therapeutic effect in OGTT was significantly reduced 1 wk after ceasing administration (Fig 3B).

These results demonstrate that upon reaching an age beyond 15 wks, *ALAS1* heterozygous mice develop impaired glucose tolerance and insulin resistance, the pathology of which is dependent on ALA levels.

### Glucagon levels, gluconeogenic gene expression and insulin signaling in aged *A1*^+/-^s

Fasting blood glucose and serum glucagon levels of aged *A1*^+/-^s were similar to WTs (Fig 2A and Table 2). The hepatic expression of genes encoding the gluconeogenic enzymes PEPCK and G6Pase were analyzed by Q-PCR revealing increased *PEPCK* mRNA levels, but reduced *G6Pase* mRNA levels in aged *A1*^+/-^s under normal dietary conditions (Table 2). In ALA-administered aged *A1*^+/-^s, the expression of both *PEPCK* and *G6Pase* were restored to normal levels (Table 2). Since G6Pase catalyzes the final step in gluconeogenesis, reduced levels of *G6Pase* mRNA could not account for the elevated blood glucose levels seen in *A1*^+/-^s, and imply that the abnormal expression of gluconeogenic genes is not relevant to the impaired glucose tolerance and insulin resistance phenotype.

**Table 2:** Expression of glucagon and neoglucogenic genes in aged *A1*^+/-^s.

Parameters	WT (*P-value vs A1*^+/-^)	*A1*^+/-^	ALA-adm. *A1*^+/-^ (*P-value vs A1*^+/-^)
Serum glucagon (pg/ml)	249.22±36.76	227.80±30.06	N.D.
	(0.328), (n = 12)	(n = 12)	
*Pepck / β-actin*	1.00±0.17	1.70±0.27	0.87±0.34
(Relative expression)	(0.036*), (n = 4)	(n = 4)	(0.052), (n = 4)
*G6Pase / β-actin*	1.00±0.19	0.42±0.10	0.98±0.34
(Relative expression)	(0.029*), (n = 4)	(n = 4)	(0.116), (n = 4)

Serum glucagon levels of aged WTs (WT) and *A1*^+/-^s under normal fed conditions (n = 12 per group). mRNA expression levels of gluconeogenic genes, *PEPCK* and *G6Pase* in the liver of aged WTs, *A1*^+/-^s and ALA-administered *A1*^+/-^s (ALA-adm. *A1*^+/-^) (n = 4 per group). Values are means ± s. e. m. for the indicated number of measurements. Statistical significance was determined by 2-tailed unpaired Student’s *t*-test

*P<0.05.

**Fig 4:**
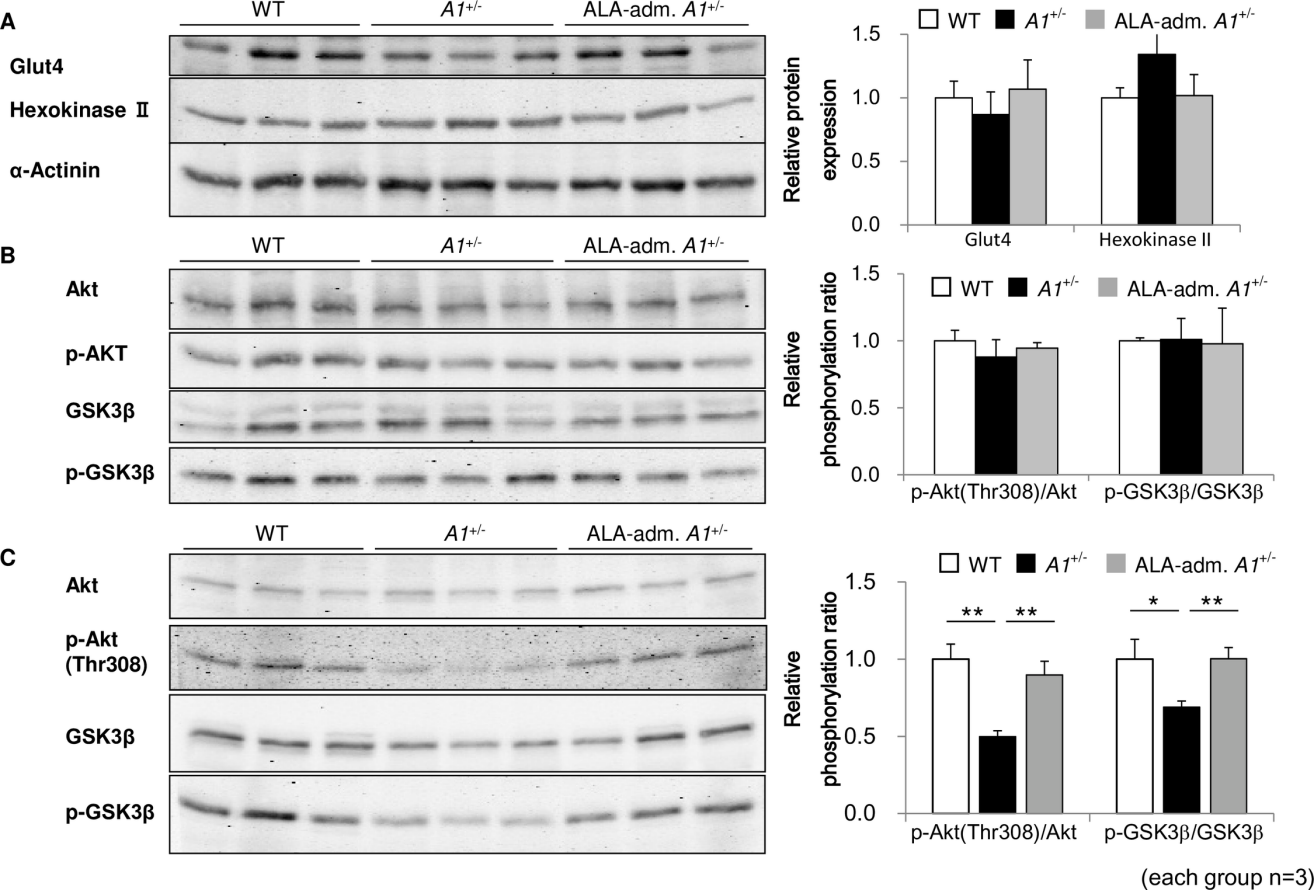
Modest reduction in insulin signaling in the skeletal muscle of aged *A1*^+/-^s. (**A**) Protein expression levels of glucose transporter Glut4 and Hexokinase II in skeletal muscle 15’ after insulin administration. (**B**,**C**) Expression levels of total or phosphorylated proteins of insulin signal-related proteins (Akt, GSK3β) in skeletal muscle 15’ after insulin administration (**B**) or under normal fed conditions (**C**) with or without ALA administration for 1 wk. α-Actinin was used as a loading control. Values are means ± s. e. m. for the indicated number of measurements. Statistical significance was determined by 2-tailed unpaired Student’s t-test, *P<0.05, **P<0.01.

In skeletal muscle and adipose tissues, Glut4 is responsible for insulin-dependent glucose uptake, which is followed by glucose conversion to glucose 6-phosphate by the enzyme hexokinase II [30]. We examined the protein levels of Glut4 and hexokinase II in skeletal muscle 15-minutes after insulin injection but found no significant differences between aged *A1*^+/-^s, WTs and ALA-administered *A1*^+/-^s (Fig 4A). Protein expression levels and the ratio of non-phosphorylated to phosphorylated forms of insulin signaling molecules such as Akt and GSK3β were also examined in skeletal muscle 15-minutes after insulin-injection, but no remarkable changes were observed between aged WTs, *A1*^+/-^s and ALA-administered *A1*^+/-^s (Fig 4B). Furthermore, under normal fed conditions, the ratios of phosphorylated to non-phosphorylated forms of Akt and GSK3β were reduced in skeletal muscle of aged *A1*^+/-^s (Fig 4C). These data suggest that ALA deficiency may affect the basal level of insulin signaling rather than the induced insulin signaling found at high serum insulin levels.

### Differentiated C2C12 myocytes with a knockdown of ALAS1 show decreased glucose uptake in response to insulin

To confirm that a deficiency in *ALAS1* can lead to impaired glucose metabolism in a cell-autonomous manner, we established a stable C2C12 myoblast cell line expressing a shRNA against *ALAS1* (termed *ALAS1*-shRNA-C2C12s). *ALAS1*-shRNA-C2C12s displayed a 60% decrease in *ALAS1* mRNA expression relative to parental C2C12 cells (S4 Fig in Supporting Information), and therefore approximately mirror the decreased expression seen in the *ALAS1* heterozygous knockout animals (Fig 1A).

**Fig 5:**
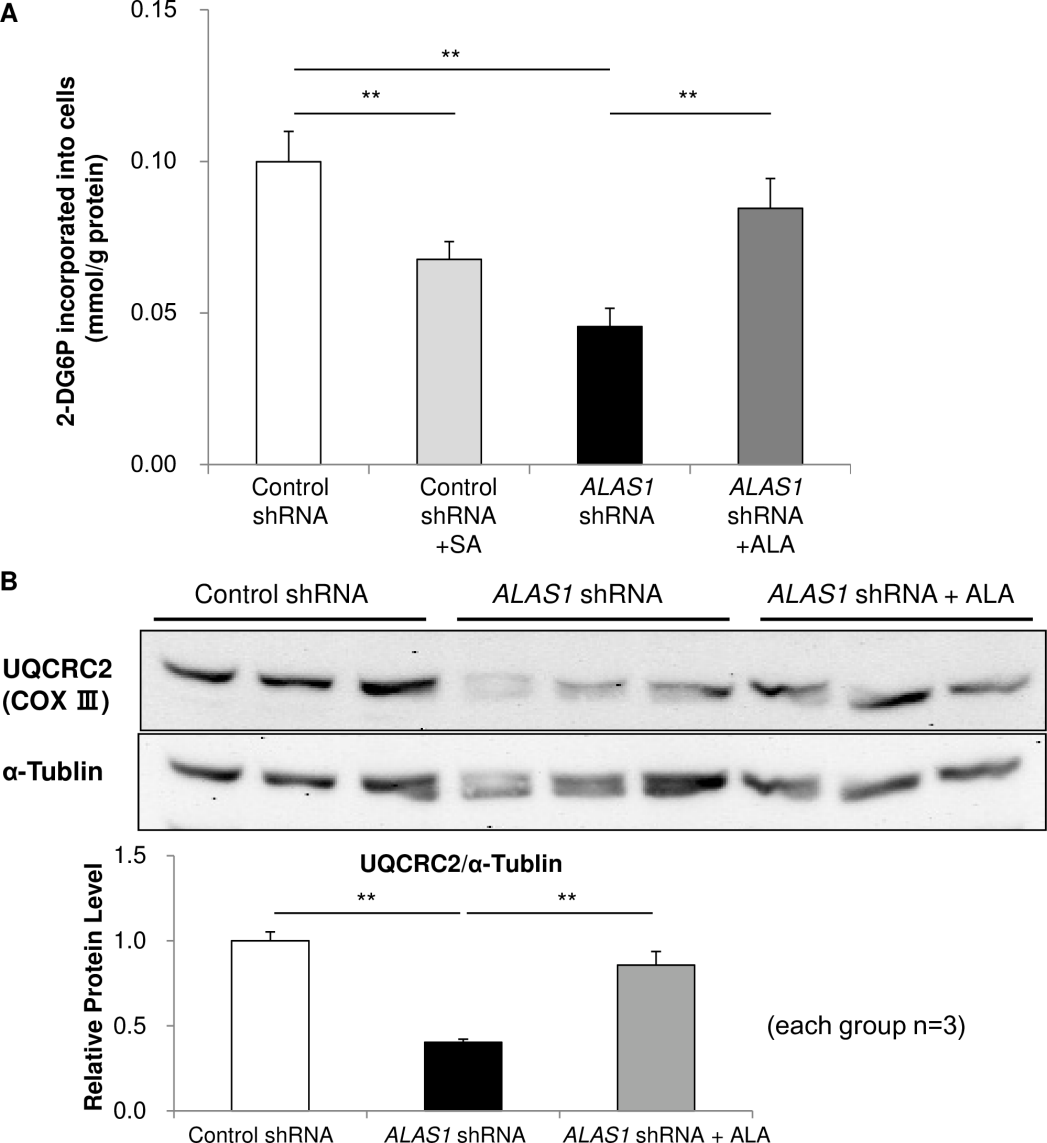
Reduced cellular glucose uptake in response to insulin and decreased expression of a mitochondrial ETC complex subunit in *ALAS1*-knockdown C2C12 myocytes. Insulin-induced glucose uptake (**A**) in differentiated control-shRNA-C2C12s with or without 50 μM succinylacetone (SA) treatment and *ALAS1*-shRNA-C2C12s with or without 100 μM ALA treatment for 1 day (n = 4–8 per condition). (**B**) Protein expression levels of UQCRC2 (Complex III) in differentiated control-shRNA- and *ALAS1*-shRNA-C2C12 myocytes under normal culture condition (n = 3 per condition). Values are means ± s. e. m. of the indicated number of measurements. Statistical significance was determined by 2-tailed unpaired Student’s *t*-test, **P<0.01.

Myocyte-differentiated *ALAS1*-shRNA-C2C12s showed reduced cellular glucose uptake in response to insulin compared to those expressing control-shRNA (termed control-shRNA-C2C12s) (Fig 5A). Significantly, ALA-treatment of myocyte-differentiated *ALAS1*-shRNA-C2C12s led to a recovery in cellular glucose uptake induced by insulin (Fig 5A). These data reveal that the defects in insulin-induced glucose uptake seen in *ALAS1*-shRNA-C2C12s are consistent with the impaired glucose tolerance and insulin resistance seen in aged *A1*^+/-^s, and demonstrate that ALA deficiency causes impaired glucose metabolism in skeletal muscle in a cell-autonomous manner.

### Heme is responsible for the impaired glucose metabolism under *ALAS1* deficiency

To determine whether ALA itself or heme impacts upon glucose metabolism, we investigated the effect of succinylacetone (SA)—an inhibitor of 5-aminolevulinate dehydratase [31]—on cellular glucose uptake in response to insulin treatment of differentiated C2C12 myocytes. SA treatment decreased cellular glucose uptake in response to insulin in differentiated control-shRNA-C2C12s (Fig 5A). The data indicate that heme influences glucose metabolism in myocytes, rather than ALA.

### Mitochondrial function is attenuated specifically in skeletal muscle under ALA deficiency

Heme is an essential molecule for mitochondrial function, being a component of several hemoproteins of the ETC [10]. It is likely that heme deficiency leads to mitochondrial dysfunction, which many previous studies have suggested is associated with insulin resistance [32–34]. Therefore, we examined whether mitochondrial dysfunction could be a contributory factor to insulin resistance in *A1*^+/-^s.

**Fig 6:**
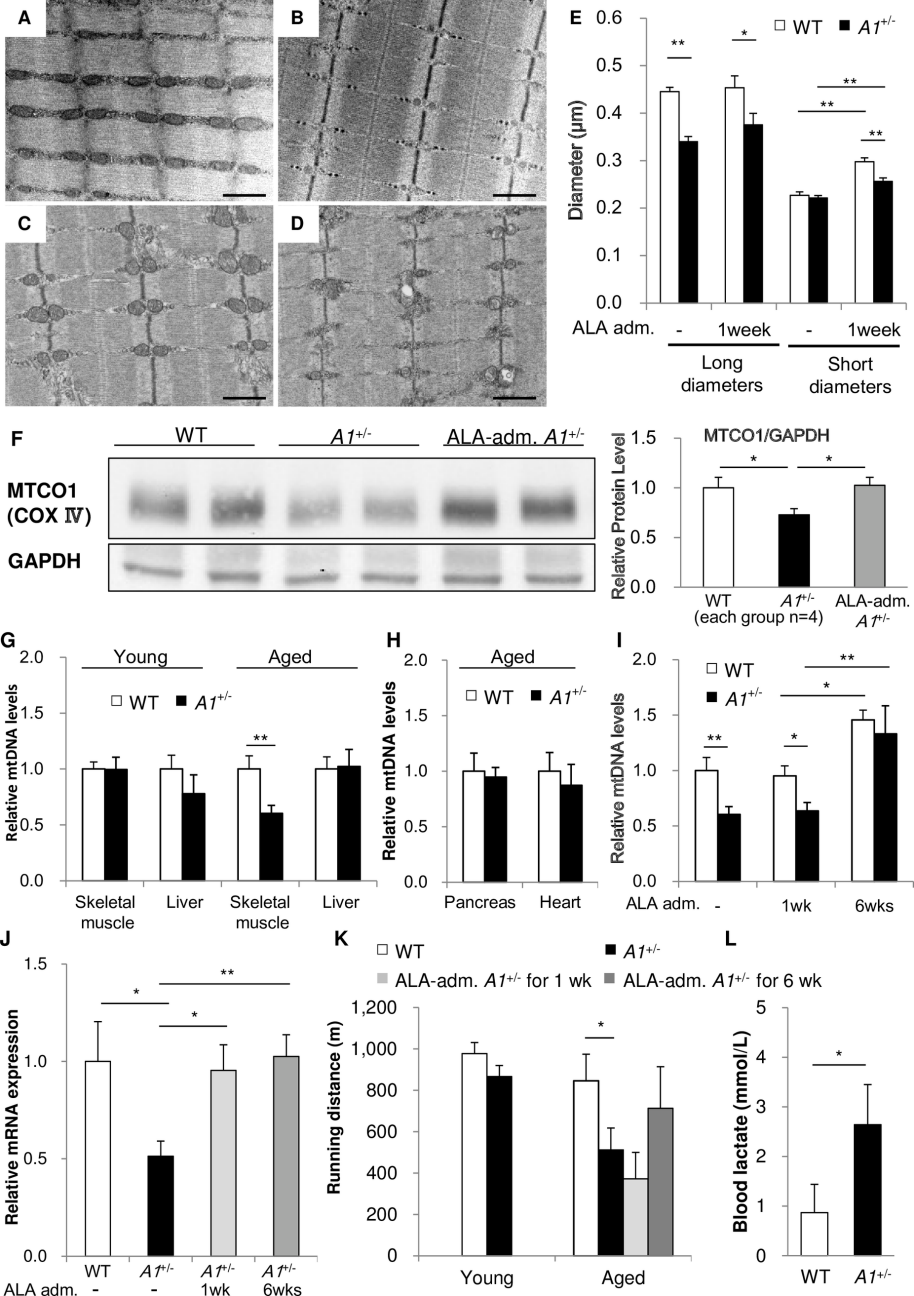
Mitochondrial morphology and number in skeletal muscle of aged *A1*^+/-^s. (**A-D**) TEM of skeletal muscle from aged WTs (**A,C**) and *A1*^+/-^s (**B,D**) without (**A,B**) or with ALA administration for 1 week (**C,D**). Magnification x20K, bar = 1 μm. (**E**) The size of mitochondria in skeletal muscle of aged WT and *A1*^+/-^s with or without ALA administration for 1 week. Longitudinal and transverse diameters of mitochondria were measured in the TEM images (measurements taken from 40–50 mitochondria). (**F**) Protein expression levels of MTCO1, a subunit of ETC complex IV, in the skeletal muscle of aged WTs, *A1*^+/-^s and *A1*^+/-^s administered ALA for 1 wk. (**G-I**) Mitochondrial DNA levels in skeletal muscle and liver of young or aged WTs or *A1*^+/-^s (**G**), in the pancreas and the heart of aged WTs and *A1*^+/-^s (**H**), and in skeletal muscle of aged WTs or *A1*^+/-^s administered ALA for 1 or 6 wk (ALA-adm. *A1*^+/-^s for 1wk or 6wks) (**I**) (n = 4–8 per group). (**J**) *PGC1a* mRNA levels in skeletal muscle of aged WTs, *A1*^+/-^s, *A1*^+/-^s administered ALA for 1 or 6 wk (n = 4–9 per group). (**K**) Running distances of treadmill tested young- or aged-WTs and *A1*^+/-^s and aged *A1*^+/-^s administered ALA for 1 wk or 6 wks (n = 10 per group). (**L**) Serum lactate levels after treadmill test of aged WTs and *A1*^+/-^s (n = 7 per group). Values are means ± s. e. m. for the indicated number of measurements. Statistical significance was determined by 2-tailed unpaired Student’s *t*-test, *P<0.05, **P<0.01, *** P<0.001.

TEM analyses of mitochondria from the skeletal muscle of aged WTs (Fig 6A) and aged *A1*^+/-^s (Fig 6B) revealed that ALA deficiency is associated with an atrophied mitochondrial morphology, a decreased electron density, and an indistinct cristae structure, suggesting that aged *A1*^+/-^s experience mitochondrial dysfunction. TEM images show that mitochondria from skeletal muscle of aged *A1*^+/-^s were smaller than those of aged WTs (Fig 6E). Significant reduction in the expression of MTCO1, a subunit of ETC complex IV, was also observed by Western blot in the skeletal muscle of aged *A1*^+/-^s compared to aged WTs (Fig 6F). To evaluate mitochondrial number and/or volume, we examined mitochondrial DNA levels in the skeletal muscle and liver of young and aged mice (Fig 6G). The mitochondrial DNA content of skeletal muscle in aged *A1*^+/-^s was approximately half that of aged WTs, while normal mitochondrial DNA levels were found in young *A1*^+/-^s, which showed no insulin resistance (Fig 2F). The hepatic mitochondrial DNA content of young and aged *A1*^+/-^s showed no significant change (Fig 6G), and the mitochondrial DNA content in the heart and the pancreas of aged *A1*^+/-^s were also normal (Fig 6H).

Mitochondrial biogenesis in skeletal muscle is regulated by PGC1α through the nuclear respiratory factors (NRFs) and the mitochondrial transcription factor A (Tfam) [35]. *PGC1α* mRNA levels in skeletal muscle from aged *A1*^+/-^s was reduced compared to aged WTs (Fig 6J), reflecting the differences seen in mitochondrial DNA (Fig 6G). Decreased expression of UQCRC2, a subunit of ETC complex III, was found in differentiated *ALAS1*-shRNA-C2C12s (Fig 5B).

To access mitochondrial function in the skeletal muscle of *A1*^+/-^s, exercise endurance was evaluated by treadmill test (Fig 6K). Young *A1*^+/-^s were capable of running similar distances until exhaustion as young WTs, whereas aged *A1*^+/-^s showed impaired physical endurance compared to aged WTs and young *A1*^+/-^s. In addition, the forced running of aged *A1*^+/-^s resulted in increased amounts of serum lactate levels relative to aged WTs (Fig 6L). However, histological examinations of skeletal muscle showed no distinguishable differences between aged *A1*^+/-^s and aged WTs when stained for cytochrome c oxidase (COX), Succinate dehydrogenase (SDH), and NADH-tetrazolium reductase (NADH-TR), respectively (data not shown).

### The effects of ALA treatment on mitochondrial defects in *A1*^+/-^s

The analyses of mitochondrial DNA and physical endurance in *A1*^+/-^s revealed an age-related onset of mitochondrial attenuated function (Fig 6G and 6K) in addition to impaired glucose tolerance and insulin resistance. Many studies have previously suggested that defects in mitochondrial activity are closely associated with the development of T2DM [32–34], but there remains considerable controversy about whether mitochondrial dysfunction is a cause or a consequence of insulin resistance [36,37]. To determine if the attenuation of mitochondrial function is essential for the development of impaired glucose tolerance and insulin resistance in *A1*^+/-^s, we performed TEM analysis for the morphology of mitochondria of aged WTs (Fig 6C) and *A1*^+/-^s (Fig 6D) that had been administered ALA for 1 wk, and examined mitochondrial DNA levels and *PGC1α* expression in the skeletal muscle of aged *A1*^+/-^s that were administered ALA for 1 wk and for 6 wks (Fig 6I and 6J). Mitochondria from the skeletal muscle of aged *A1*^+/^s administered ALA for 1 wk showed an improvement in morphology (an appearance more similar to WT controls) relative to those of aged *A1*^+/-^s without ALA treatment (Fig 6D). In fact, the mitochondria of aged *A1*^+/-^s administered ALA for 1 wk were slightly larger than those of aged *A1*^+/-^s without ALA treatment (Fig 6E). However, they remained significantly smaller than those of aged WTs administered ALA for 1 wk (Fig 6E). ALA administration for 1 wk caused an increase in the width diameters of mitochondria both of WTs and *A1*^+/-^s (Fig 6E). While after 6 wks of ALA administration, both aged WTs and *A1*^+/-^s showed increased mitochondrial DNA in skeletal muscle relative to non-treated animals, there was no observable change after only 1 wk of treatment (Fig 6I). The levels of *PGC1α* mRNA and of MTCOI protein (a Complex IV subunit) were significantly increased in the skeletal muscle of aged *A1*^+/-^s after administration of ALA for 1 wk (Fig 6F and 6J) and 6 wks (Fig 6J), but the physical endurance of the aged *A1*^+/-^s remained impaired after ALA administration for 1 wk, and required 6 wks of ALA treatment to restore endurance capacity to normal levels (Fig 6K). These data suggest that in aged *A1*^+/-^s, ALA administration for 1 wk can partially improve aberrant mitochondrial morphology and restore *PGC1a* expression but there remains a sustained decrease in mitochondrial DNA levels and an impaired physical endurance capacity in the animals. These results are particularly noteworthy, since despite an incomplete restoration of mitochondrial function in aged *A1*^+/-^s it was possible to observe a full recovery form impaired glucose tolerance and insulin resistance after a 1 wk period of ALA treatment, (Figs 3B, 3C, 6C–6F, 6I, 6J and 6K). It may be concluded therefore, that complete reversal of the attenuated mitochondrial phenotype in aged *A1*^+/-^s is not necessary for the recovery from impaired glucose tolerance and insulin resistance.

## Discussion

In this study, we demonstrate that *A1*^+/-^s develop impaired glucose tolerance, insulin resistance and an attenuation of mitochondrial function in skeletal muscle beyond 15 wks of age, correlating with the reduced expression of *ALAS1* (Fig 2B, 2C, 2F and 2G). Oral administration of ALA for 1 wk fully reversed impaired glucose tolerance and insulin resistance in aged *A1*^+/-^s (Fig 3B and 3C), contrasting to minimal effects on glucose tolerance in WTs (Fig 3A). Therefore, this study provides the first evidence that an *in vivo* deficiency of ALA can lead to impaired glucose tolerance and insulin resistance, and reveals an unexpected link between heme and glucose metabolism.

Our data further indicate that the impairment of glucose metabolism caused by ALA deficiency occurs in a cell-autonomous manner. In this regard, knockdown of *ALAS1* in C2C12 myocytes caused a decrease in glucose uptake in response to insulin (Fig 5A), providing a molecular mechanism by which heme regulates cellular glucose metabolism. It may be unexpected that a halving in *ALAS1* mRNA expression could result in impaired glucose metabolism because it had been reported that ALAS1 expression is regulated by heme in a negative feedback manner at multiple steps such as *ALAS1* mRNA degradation [38], ALAS1 translation [39], and cellular localization of ALAS1 [40], as well as *ALAS1* transcription [41]. In fact, we had performed heme quantification in skeletal muscle several times, but could not observe any significant reduction in heme content in aged *A1*^+/-^s (S1 Fig in Supporting Information). In skeletal muscle, most of cellular heme is expected to bind to apomyoglobin, while a small fraction of cellular heme, that is weakly protein-bound heme, can act as the “regulatory free heme pool” [42]. Therefore, it is probable that the approximate halving in *ALAS1* mRNA levels reduces “the regulatory heme pool” in skeletal muscle. We speculate that the ALA deficiency in aged *A1*^+/-^s substantially affects the size of the “regulatory free heme pool” rather than total cellular heme. It is noteworthy that aged *A1*^+/-^s showed no abnormalities in iron metabolism (S2A–S2D Fig in Supporting Information) and co-treatment of iron together with ALA had no additional effect on glucose tolerance in the animals (Fig 3D), suggesting that iron metabolism is not relevant to the impaired glucose metabolism in aged *A1*^+/-^s.

Numerous studies have suggested that mitochondrial dysfunction in skeletal muscle could be related to insulin resistance, but until now it has remained unclear whether mitochondrial dysfunction could be a causative factor in insulin resistance [32–34,36,37]. The data from ALA-treated *A1*^+/-^s (Figs 3 and 6) suggest that the attenuation of mitochondrial function, impaired glucose tolerance and insulin resistance emerge coincidentally, but the conditions are not fully co-dependent on one another since a marked improvement in impaired glucose tolerance and insulin resistance was observed after only 1 wk of ALA-treatment, contrasting with only a partial improvement in mitochondrial morphology, levels and *PGC1α* expression (Fig 6E and 6J). In fact, the decreased mitochondrial DNA levels and impaired physical endurance observed in *A1*^+/-^s, appear to be secondary to impaired glucose tolerance and insulin resistance and required 6 wks of ALA treatment to observe a reversal in the defects (Fig 6I and 6K). The ALA administration studies show that recovery from the decreased levels of mitochondrial DNA is preceded by a full restoration of *PGC1α* expression in *A1*^+/-^s (Fig 6I and 6J). PGC1α directly promotes *NRF1* expression, which in turn upregulates *Tfam* to stimulate mitochondrial DNA replication [43]. Therefore, it is probable that the increase in mitochondrial DNA levels follows upregulation of *PGC1α* expression, which had been described previously in serum-stimulated quiescent BALB/3T3 fibroblasts [43].

PGC1α has been suggested to act as both a master regulator of heme biosynthesis [14] and of mitochondriogenesis [44,45]. PGC1α upregulates *ALAS1* transcription through the transcription factors FOXO1 and NRF1 [14]. The transcriptional repressor, Rev-erbα is known as a physiological receptor for heme [15–17], and Wu *et al*. have proposed that heme-binding to Rev-erbα inhibits *PGC1α* transcription to downregulate *ALAS1* and to form a heme-dependent negative feedback loop in the liver [44]. According to this theory, *ALAS1* expression in *A1*^+/-^s would be kept at normal levels by increased PGC1α. However, in actuality, *A1*^+/-^s showed decreased *ALAS1* and *PGC1α* expression (Figs 1A, 1B and 6J), and ALA administration increased *PGC1α* expression in skeletal muscle of *A1*^+/-^s (Fig 6J). Therefore, we posit the existence of a putative molecular mechanism by which heme regulates *PGC1α* expression in skeletal muscle without involving Rev-erbα because heme repletion is indispensable to normal PGC1α expression and mitochondriogenesis (Fig 6I and 6J). In addition, the observation that normal levels of mtDNA were found in the liver of aged *A1*^+/-^s (Fig 6G) could be accounted for by the heme-dependent negative feedback loop on *PGC1α* transcription [44], wherein decreased heme levels causes a release in *PGC1α* control.

ALA administration stimulated mitochondrial morphology and mitochondrial DNA levels of both WTs and *A1*^+/-^s (Fig 6E and 6I). Recently, it has been reported that human sarcopenia can be improved by ALA administration [46]. Heme repletion through ALA administration might stimulate mitochondriogenesis through PGC1α activation in skeletal muscle similar to what was observed in ALA-administered *A1*^+/-^s (Fig 6J).

From cohort studies, oral ALA supplementation led to improvements in prediabetic [12] and type-2 diabetic [11] patients. It is unlikely that heme deficiency is exclusively caused by defects in ALAS1 protein production. Rather, aging [47,48] and other situations are likely to result in heme deficiency more commonly than expected. Considering that aged *A1*^+/-^s manifest insulin resistance and aged rats have lower activity of hepatic ALAS as compared to young animals [48], age-related insulin resistance might be associated with heme deficiency.

We found no evidence of abnormal insulin signaling in the skeletal muscle of aged *A1*^+/-^s after insulin injection (Fig 4B). Insulin signal transduction under basal, normal fed conditions was slightly impaired in aged *A1*^+/-^s (Fig 4C), however this is unlikely to be a causative factor for impaired glucose tolerance and insulin resistance. At present, the mechanism by which ALA/heme deficiency causes impaired glucose metabolism remains unclear, but we believe that the abnormalities in aged *A1*^+/-^s discovered in this study provide a novel clue for understanding an unknown regulatory mechanism for glucose metabolism.

## Supporting information

S1 Fig Heme content in the mitochondrial and cytosolic fraction of skeletal muscle. Heme content in mitochondrial and cytosolic fraction of skeletal muscle in aged WTs and *A1*^+/-^s under normally feeding conditions (n = 3–5 per group).
(TIF)

S2 Fig **Serum iron levels, total iron binding capacity (TIBC), unsaturated iron binding capacity (UIBC) and serum ferritin levels** (**A-D**) Serum iron levels (**A**), TIBC (**B**), UIBC (**C**) and serum ferritin levels (**D**) in aged *A1*^+/-^s and WTs under normally feeding condition (n = 6 per group).
(TIF)

S3 Fig The effects of ALA treatment duration and dose on impaired glucose metabolism in aged *A1*^+/-^s. (**A,B**) Blood glucose levels after glucose load (OGTT) in aged *A1*^+/-^s following oral administration of ALA (200 mg/kg BW/day) for 1 wk or 2 wks (**A**) or of ALA (200 mg or 400 mg/kg BW/day) for 1 wk (**B**) (n = 5 per group). (**C**) Blood glucose levels after insulin injection (ITT) in aged *A1*^+/-^s after oral administration of a lower dose of ALA (200 mg/kg BW/day) for 1 wk. Values are means ± s. e. m. for the indicated number of measurements. Statistical significance was determined by 2-tailed unpaired Student’s t-test, *P<0.05, **P<0.01, *** P<0.001.
(TIF)

S4 Fig *ALAS1* mRNA expression levels in control- and *ALAS1*-shRNA-C2C12 myoblasts. The relative levels of *ALAS1* mRNA were measured by Q-PCR in differentiated control- and *ALAS1*-shRNA C2C12 cells (n = 4 per condition). Values are mean ± s. e. m. of the indicated number of measurements. Statistical significance was determined by 2-tailed unpaired Student’s *t*-test, *P<0.05.
(TIF)
