# Supplementary figures and images for: 5-aminolevulinic acid (ALA) deficiency causes impaired glucose tolerance and insulin resistance coincident with an attenuation of mitochondrial function in aged mice
Source: PLoS One. 2018 Jan 24;13(1):e0189593. doi: 10.1371/journal.pone.0189593 (PMC5783358; doi:10.1371/journal.pone.0189593)

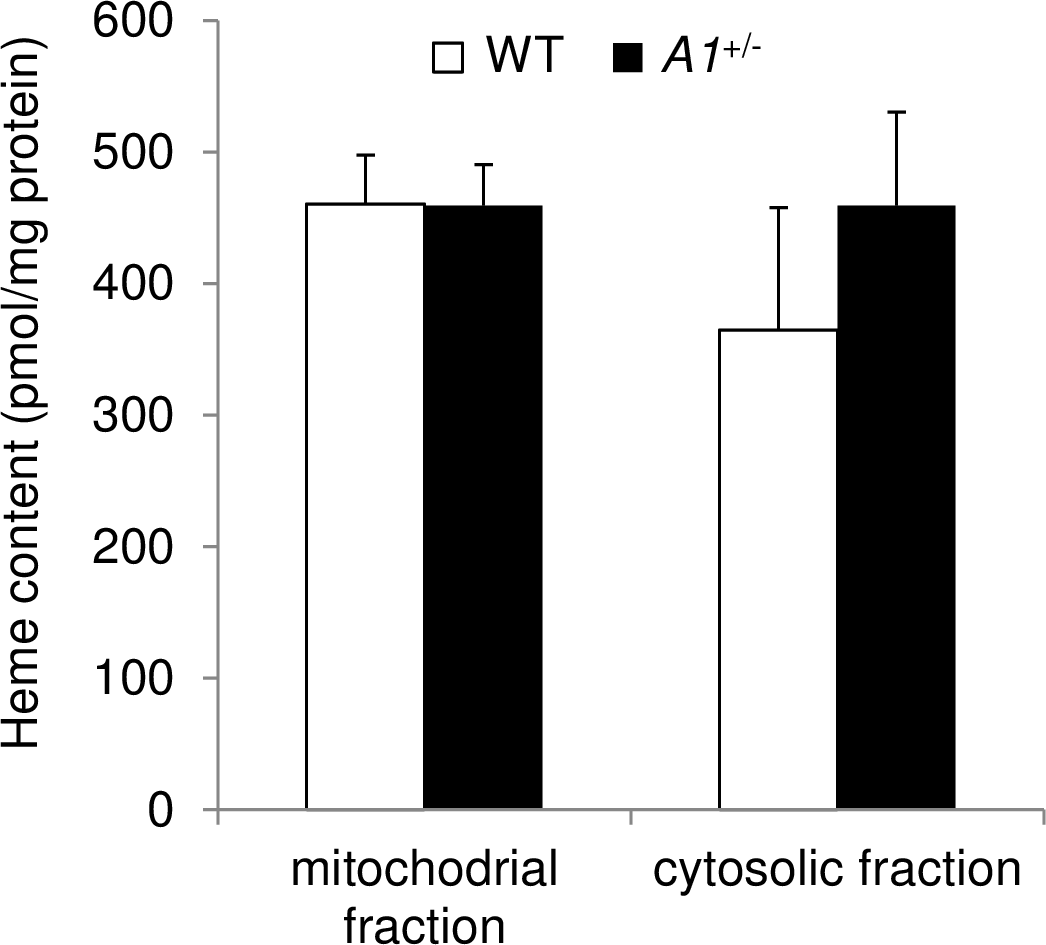

Supplement: S1 Fig — Heme content in mitochondrial and cytosolic fraction of skeletal muscle in aged WTs and A1+/-s under normally feeding conditions (n = 3–5 per group). (TIF) [file pone.0189593.s001.tif]

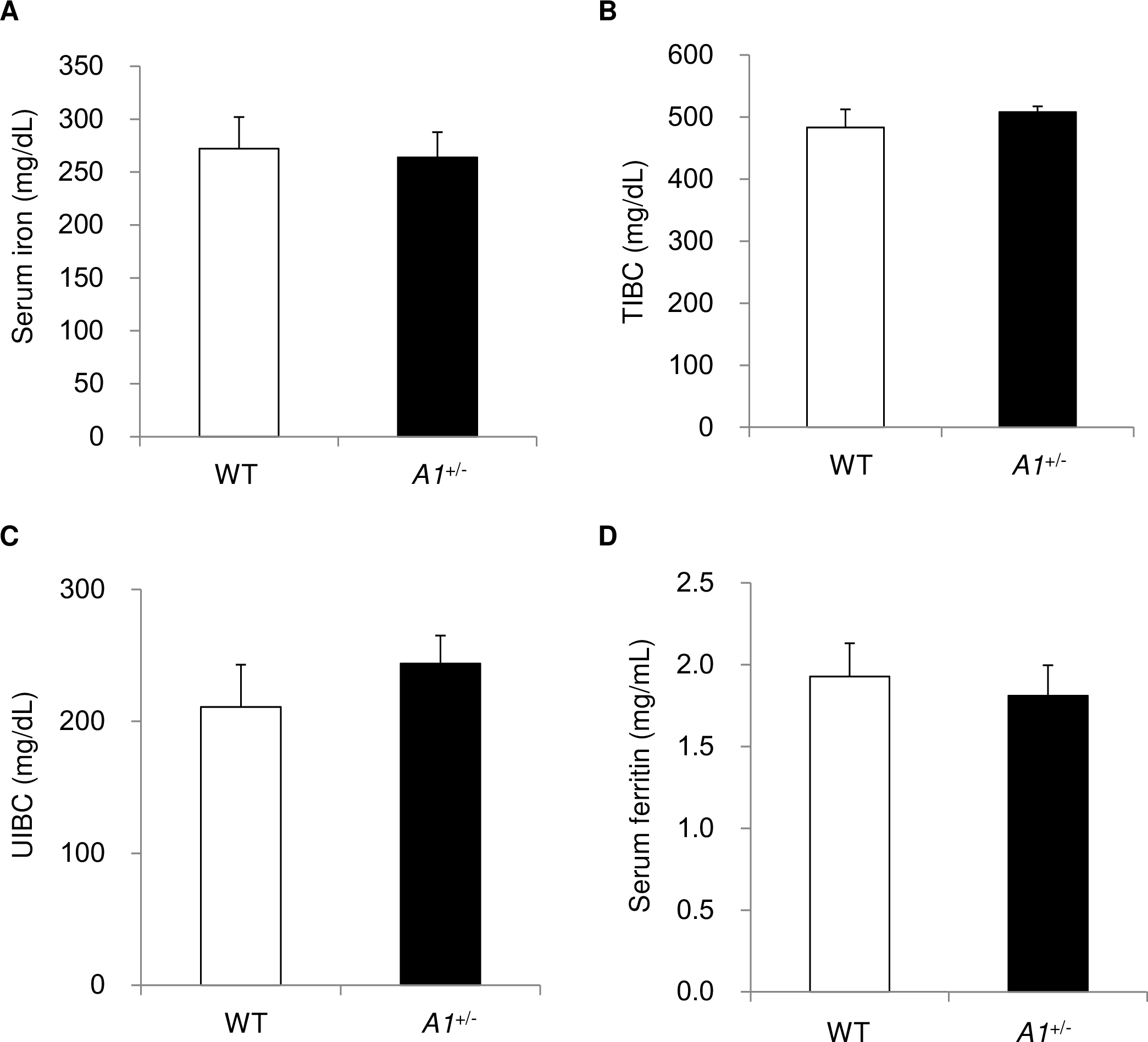

Supplement: S2 Fig — Serum iron levels, total iron binding capacity (TIBC), unsaturated iron binding capacity (UIBC) and serum ferritin levels (A-D) Serum iron levels (A), TIBC (B), UIBC (C) and serum ferritin levels (D) in aged A1+/-s and WTs under normally feeding condition (n = 6 per group). (TIF) [file pone.0189593.s002.tif]

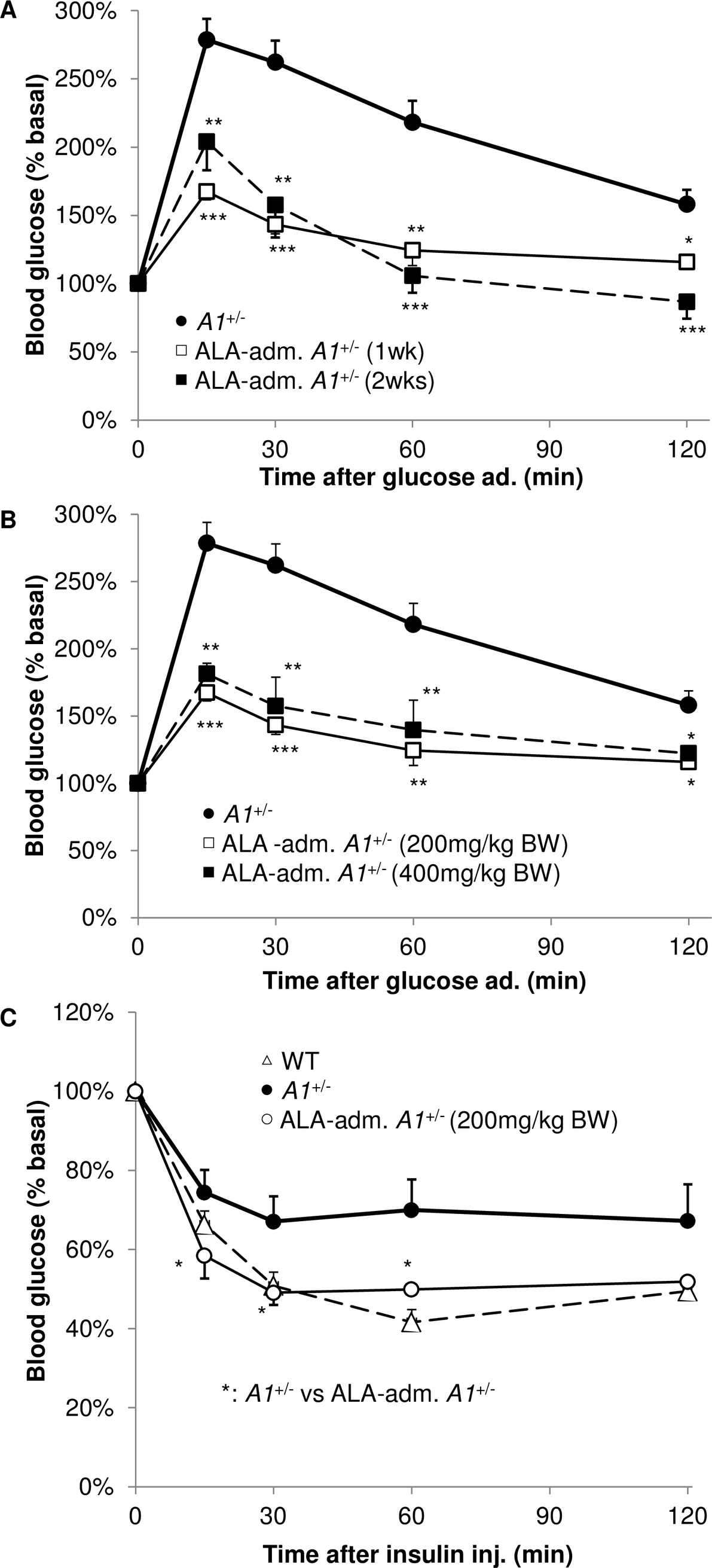

Supplement: S3 Fig — (A,B) Blood glucose levels after glucose load (OGTT) in aged A1+/-s following oral administration of ALA (200 mg/kg BW/day) for 1 wk or 2 wks (A) or of ALA (200 mg or 400 mg/kg BW/day) for 1 wk (B) (n = 5 per group). (C) Blood glucose levels after insulin injection (ITT) in aged A1+/-s after oral administration of a lower dose of ALA (200 mg/kg BW/day) for 1 wk. Values are means ± s. e. m. for the indicated number of measurements. Statistical significance was determined by 2-tailed unpaired Student’s t-test, *P<0.05, **P<0.01, *** P<0.001. (TIF) [file pone.0189593.s003.tif]

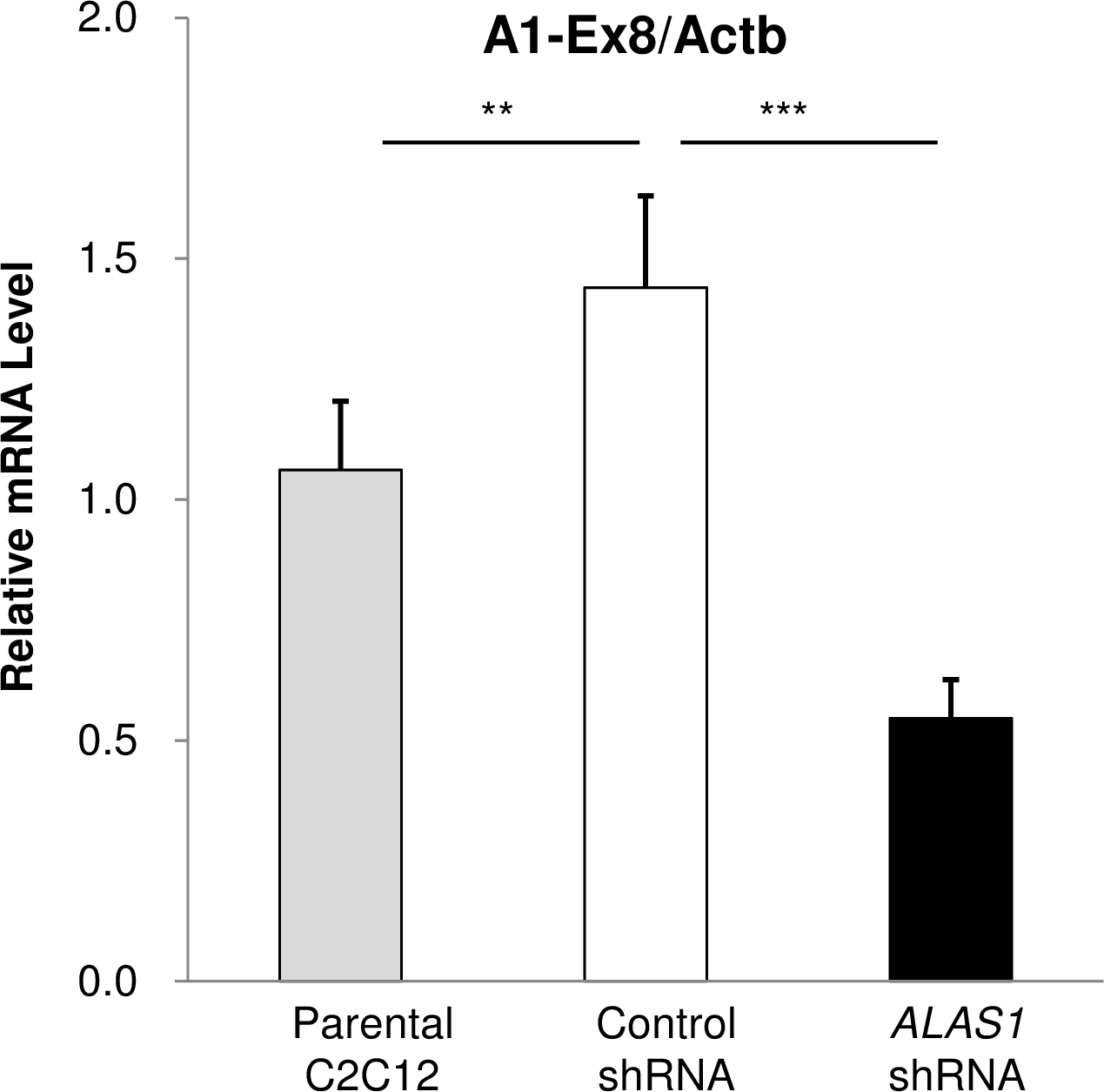

Supplement: S4 Fig — The relative levels of ALAS1 mRNA were measured by Q-PCR in differentiated control- and ALAS1-shRNA C2C12 cells (n = 4 per condition). Values are mean ± s. e. m. of the indicated number of measurements. Statistical significance was determined by 2-tailed unpaired Student’s t-test, *P<0.05. (TIF) [file pone.0189593.s004.tif]
